# Meta-analytic evidence for a sex-diverging association between alcohol use and body mass index

**DOI:** 10.1038/s41598-022-25653-w

**Published:** 2022-12-19

**Authors:** Eva-Maria Siegmann, Massimiliano Mazza, Christian Weinland, Falk Kiefer, Johannes Kornhuber, Christiane Mühle, Bernd Lenz

**Affiliations:** 1 Department of Psychiatry and Psychotherapy, University Hospital Erlangen, Friedrich-Alexander University Erlangen-Nürnberg (FAU), Schwabachanlage 6, 91054 Erlangen, Germany; 2 Department of Addictive Behavior and Addiction Medicine, Central Institute of Mental Health (CIMH), Medical Faculty Mannheim, Heidelberg University, Mannheim, Germany

**Keywords:** Addiction, Lifestyle modification, Weight management, Nutrition

## Abstract

Alcohol use is an important health issue and has been suggested to contribute to the burden produced by obesity. Both alcohol use and obesity are subject to sex differences. The available studies on the relationship between alcohol use and body mass index (BMI) report inconsistent results with positive, negative, and null findings which requests a meta-analytic approach. Therefore, we conducted a meta-analysis of case–control, cohort, and cross-sectional studies. The systematic literature search and data extraction was performed by 3 independent raters. We conducted sex-separated meta-analyses and -regressions to investigate how alcohol consumption associates with BMI. Our systematic literature search resulted in 36 studies with 48 data sets (N_men_ = 172,254; k_men_ = 30; N_women_ = 24,164; k_women_ = 18; N_unknown sex_ = 672,344; k_unknown sex_ = 24). Alcohol use was associated with higher BMI in men (g = 0.08 [0.07; 0.09]) and lower BMI in women (g = − 0.26 [− 0.29; − 0.22]). Moreover, we found the amount of daily alcohol intake in men (β = 0.001 [0.0008; 0.0014]) and ethnicity in women (g[Caucasians] = − 0.45 versus g[Asians] = − 0.05; z = 11.5, p < 0.0001) to moderate these effects. We here identified sex-diverging relationships between alcohol use and BMI, found daily alcohol intake and ethnicity to sex-specifically moderate these effects, and argue that sex-specific choice of beverage type and higher amount of daily alcohol use in men than in women account for these observations. Future research is needed to provide empirical evidence for the underlying mechanisms.

## Introduction

For 2016, the World Health Organization (WHO)^1^ estimates a total alcohol per capita consumption of 6.4 L. Harmful use of alcohol accounted for approximately 3 million deaths (= 5.3% of all deaths) and 132.6 million disability-adjusted life years (DALYs) (= 5.1% of all DALYs). Moreover, high prevalence rates for harmful alcohol use and dependence of 8.8% and 8.2% are estimated in the WHO European Region and the WHO Region of the Americas. Women vary from men in their alcohol use patterns and risk of alcohol use disorder (AUD); also, sex differences in mechanisms underlying alcohol use and AUD have been reported^2–16^.

Obesity is a health concern, particularly in the Western world. As alcohol contains much energy (29 kJ per gram alcohol) and most people in Europe (59.9%) and the Americas (54.1%) drink alcohol^1^, it is speculated that alcohol contributes to the obesity problem. However, the association between alcohol intake and body mass index (BMI) is not that simple. Ethanol intake inhibits different insulin actions in the body^17^ with the possible long-term consequence of a generalized insulin resistance^17,18^ contributing to overweight. Furthermore, obesity and AUD share common etiopathogenetic mechanisms such as mesolimbic dysfunction^19–22^ with lower dopamine D2 receptor availability^23^, increased cue-reactivity^24,25^, and genetics^26,27^. Similar to alcohol use, BMI and obesity vary significantly between women and men with higher rates of obesity in the female group^28^.

A systematic review published in 2011 addressed the association between alcohol intake and gain of weight^29^. The authors’ evaluation of large cross-sectional and well-powered prospective studies with long follow-up periods revealed contradictory results. However, they assumed a positive association to be more likely in men (vs. women) and in heavy drinkers, whereas there were negative or no associations in moderate consumers. Indeed, also studies that were published more recently demonstrate positive and negative relationships as well as null findings^30–32^. Negative associations might be explained by the greater thermogenic effect of alcohol than carbohydrates or fat with a lower net efficiency^33^. Initial evidence also indicates that sex differences (e.g., in choice of beverage type and drinking patterns, which influence the energy intake) might account for the ambiguous relationship between alcohol intake and BMI^29^. A more recent systematic review and meta-analysis published in 2022 showed that alcohol consumption increased the risk of obesity in adults 2.05 times compared to non-drinkers^34^, but did not distinguish between moderate vs. heavy drinkers or men vs. women. Another recent meta-analysis that focused on overweight, obese, or abdominally obese subjects again reports higher odds of obesity and overweight in heavy drinkers, but did not include normal weight samples^35^.

Thus, we here present a systematic review and meta-analysis to investigate how alcohol consumption is related to BMI while also exploring the moderating influence of sex, the amount of alcohol consumed, the percentage of smokers, and differences between ethnicities. Since the existing literature is inconclusive, we decided to follow an exploratory approach.

## Materials and methods

### Search strategy and study selection

We conducted a two-step literature search from study inception until February 4th, 2021, using PubMed and Google Scholar including case–control, cohort, and cross-sectional studies. In the course of the review process, we updated this literature search from study inception until September 9th, 2022. Due to the enormous number of results when applying a full-text search strategy (> 1,150,000 hits), we limited our search strategy exclusively to titles. The search terms *alcohol dependence, alcohol use disorder, binge drinking, alcohol, social consumption, social drinking, withdrawal, alcohol intoxication, seizure, delirium, AUDIT, CAGE, beer, wine, liquor* were used to represent alcohol intake and were combined with the search terms *BMI* and *body mass index*. In a second step, the reference lists of retrieved articles were searched manually for further eligible titles. All abstracts were screened applying the selection criteria that are detailed in our coding protocol (Supplementary Table S1). The remaining articles were checked for eligibility according to the Preferred Reporting Items for Systemic Reviews and Meta-analyses (PRISMA) statement^36^ on the basis of a full-text review and the entire literature search was summarized according to these PRISMA guidelines^37^.

### Data extraction

The data extraction process strictly followed our approach described in Siegmann et al.^11^ and Siegmann et al.^38^ and was performed by two out of three investigators (C. M., E. S., M. M.) for different portions of the publications each. All recorded variables can be found in the previously defined coding protocol (Supplementary Table S1). Disagreement was resolved by discussion and compromise on the eventually extracted values. We assessed the risk of bias with either the Newcastle–Ottawa Scale for case–control studies^39^ or an adaptation of the Newcastle–Ottawa Scale for cohort studies^39^, which was specifically designed for cross-sectional studies by Herzog et al.^40^ in their systematic review. The final risk of bias values were obtained by averaging the extractors’ values.

### Statistical analysis

All analyses were conducted and all figures were made using the metafor package^41^ within the open-source software environment R, version 4.1.1^42^ and GraphPad Prism 8.4.3 (Graph Pad Soft- ware Inc., San Diego, CA, USA).

We estimated the standardized mean difference (Hedges’ g) in BMI among drinking subjects and control subjects and, in a second step, explored the influence of sex. Following our coding protocol (Supplementary Table S1) we distinguished between data from males, data from females, and data from studies not reporting sex-separated measures (“unknown group”).

In order to combine studies reporting different measures of effect, correlative and odds ratio data were transformed into Hedges’ g using common transformation formulas^43^. The drinking group was characterized by various measures: (1) alcohol consumption levels in grams per day, (2) blood alcohol levels, (3) frequency of alcohol drinking, (4) score in the AUDIT questionnaire^44^, (5) being diagnosed with AUD by a psychiatrist, (6) score in the Obsessive–Compulsive Drinking Scale, German version (OCDS-G)^45^, (7) frequency of hospital readmissions following withdrawal treatment, and (8) being classified as binge drinkers according to a definition by Patrick et al.^46^. The control group either consisted of never-drinkers and non-binge-drinkers or was defined as consuming < 1 drink per week, drinking < 1 day per week, or scoring below the cut-off value of the AUDIT questionnaire^44^.

Owing to our study design which compared more than one variation of drinking status (e.g., light, moderate, and heavy drinking) against a single control group, the meta-analytic effect size estimates are correlated and non-independent, respectively. To account for this repeated usage of one common control group, we performed a multivariate random-effects meta-analysis recommended for the analysis of multiple-treatment studies^47,48^. The Q-statistic is reported as a measure for heterogeneity. We decided against conventional ad-hoc approaches, e.g., averaging multiple reported effects, since these would have resulted in loss of useful information for moderator analyses^47^. We ran prespecified multivariate meta-regressions for the moderators drinking amount in grams per day, percentage of smokers in the drinking group, and study quality (i.e., the risk of bias in these studies). We also tested whether drinking amount is suited as a quadratic predictor. All meta-regressions were Bonferroni-corrected for multiple testing. Since ethnicity is a variable that influences both alcohol-related behavior^49,50^ as well as BMI or body fat^51^, we investigated the difference in the outcome measure among the ethnicities Caucasian, Asian, African, and Hispanic in a prespecified subgroup analysis. Based on the aforementioned results by Sayon-Orea et al.^29^ concerning the influence of different beverage types, we planned to run another prespecified subgroup analysis distinguishing between beer, wine, liquor, and mixed alcohol consumption.

Small study effects were assessed by visual detection of asymmetries in a contour-enhanced funnel plot^52,53^. The sensitivity of our analysis was evaluated by comparing models with and without effect sizes which we assume to be influential outliers^54^. They were detected following an exploratory data analysis^55^. p < 0.05 (2-sided) was considered statistically significant.

## Results

### Eligible studies

**Figure 1:**
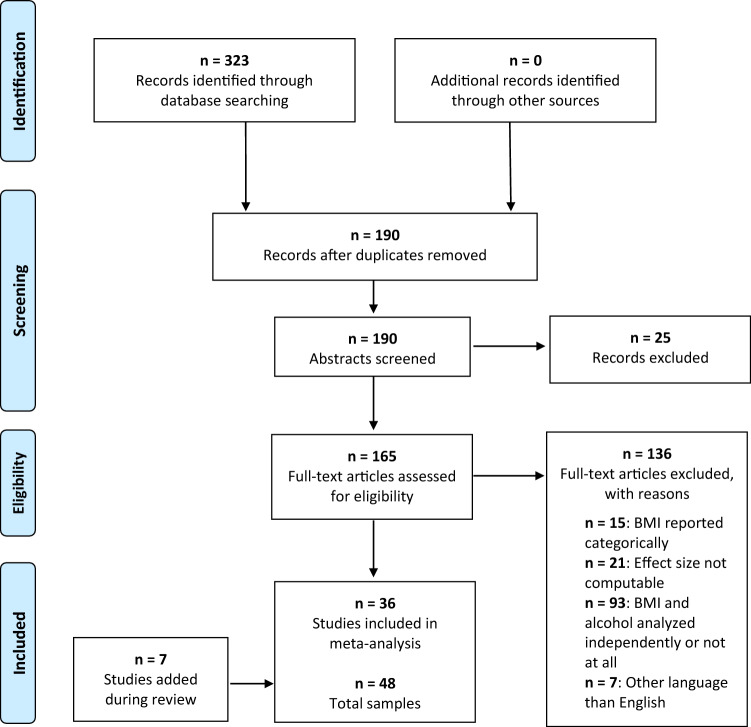
The PRISMA flow chart. This flow diagram visualizes the systematic literature search for eligible studies as well as reasons for exclusion of uneligible studies.

The literature search is summarized in the PRISMA flow chart (Fig. 1). We identified 36 articles^13,31,56–89^ comprising 48 independent samples. The characteristics of all included studies are detailed in Table 1.

**Table 1:** Characteristics of all included studies.

First author, year of publication	Case–control data								
						Drinking group		Control group	
	Country	Sex	Sample’s mean age (in years)	Study design	Mean amount of alcohol consumed (in g per day)	n	BMI (M ± SD)	n	BMI (M ± SD)
Roggi et al. 1992^74^	Italy	Male	51.8	Cross-sectional	59.5	210	26.04 ± 3.7	20	26.63 ± 4.0
Roggi et al. 1992^74^		Female	55.0		23.7	162	25.44 ± 4.6	122	26.63 ± 4.7
Männistö et al. 1996^63^ ^a,d^	Finland	Male	NA	Cross-sectional	7.5	12,850	26.17 ± NA	3045	26.03 ± NA
Männistö et al. 1996^63^ ^a,d^			NA		22.5	6019	26.45 ± NA		
Männistö et al. 1996^63^ ^a,d^			NA		45.0	4031	26.52 ± NA		
Männistö et al. 1996^63^ ^a,d^			NA		90.0	1270	26.44 ± NA		
Männistö et al. 1997^64^ ^e^	Finland	Male	45.8	Cross-sectional	14.4	367	26.10 ± NA	70	28.00 ± NA
Männistö et al. 1997^64^ ^e^						75	26.50 ± NA		
Männistö et al. 1997^64^ ^e^						228	26.90 ± NA		
Männistö et al. 1997^64^ ^e^						122	26.10 ± NA		
Männistö et al. 1997^64^ ^e^		Female	45.8		3.6	248	25.10 ± NA	174	26.60 ± NA
Männistö et al. 1997^64^ ^e^						255	24.90 ± NA		
Männistö et al. 1997^64^ ^e^						162	26.70 ± NA		
Männistö et al. 1997^64^ ^e^						146	25.30 ± NA		
Skrzypczak et al. 2008^61^	Poland	Female	56.3	Cross-sectional	NA	1579	25.26 ± 3.9	8675	27.92 ± 4.83
Kawamoto et al. 2009^83^	Japan	Male	NA	Cross-sectional	11.5	205	23.60 ± 3.1	80	23.40 ± 3.6
Kawamoto et al. 2009^83^			NA		34.4	234	23.40 ± 2.8		
Kawamoto et al. 2009^83^			NA		68.7	159	23.90 ± 3.0		
Loomba et al. 2009^82^	US	Mixed	70.0	Cross-sectional	6.0	739	25.00 ± 4.0	869	25.00 ± 4.0
Loomba et al. 2009^82^					18.0	414	25.00 ± 3.0		
Loomba et al. 2009^82^					30.0	233	25.00 ± 3.0		
Loomba et al. 2009^82^					54.0	109	26.00 ± 4.0		
Yue et al. 2012^81^	China	Male	NA	Cross-sectional	9.9	213	25.64 ± 3.2	243	25.50 ± 3.9
Yue et al. 2012^81^			NA		30.0	123	26.48 ± 3.1		
Yue et al. 2012^81^			NA		60.0	61	25.43 ± 3.2		
Ahaneku et al. 2014^57^	Nigeria	Mixed	43.8	Cross-sectional	NA	70	25.64 ± 5.9	121	24.75 ± 4.7
Shaikh et al. 2015^80^ ^f^	US	Mixed	NA	Cohort	2.6	7288	27.40 ± NA	4710	27.20 ± NA
Shaikh et al. 2015^f^			NA		14.6	3679	26.80 ± NA		
Shaikh et al. 2015^80^ ^f^			NA		36.0	1219	26.60 ± NA		
Yi et al. 2016^79^	Republic of Korea	Male	58.8	Cohort	6.7	46,503	23.70 ± 2.6	34,435	23.50 ± 2.8
Yi et al. 2016^79^					41.1	26,797	23.80 ± 2.7		
Rask-Andersen et al. 2017^78^ ^a,c^	Great Britain	Mixed	NA	Genome-wide association study (GWAS)	NA	12,966	28.09 ± 0.1	7944	28.31 ± 0.1
Rask-Andersen et al. 2017^78^ ^a,c^			NA		NA	30,412	27.57 ± 0.1		
Rask-Andersen et al. 2017^78^ ^a,c^			NA		NA	27,250	27.08 ± 0.1		
Rask-Andersen et al. 2017^78^ ^a,c^			NA		NA	24,424	26.84 ± 0.1		
Cho et al. 2018^e^	Republic of Korea	Mixed	15.2	Cross-sectional	NA	15	22.32 ± NA	520	21.18 ± NA
Booranasuksakul et al. 2019^77^	Thailand	Male	NA	Cross-sectional	6.1	69	21.43 ± 3.5	44	20.78 ± 4.12
Booranasuksakul et al. 2019^77^			NA		29.9	12	21.76 ± 4.1		
Booranasuksakul et al. 2019^77^			NA		208.7	19	23.11 ± 3.1		
Booranasuksakul et al. 2019^77^		Female	NA		6.2	98	21.34 ± 3.7	123	20.74 ± 3.9
Booranasuksakul et al. 2019^77^			NA		29.9	12	23.34 ± 3.7		
Booranasuksakul et al. 2019^77^			NA		199.8	19	22.49 ± 3.5		
Nishigaki et al. 2020^76^	Japan	Male	NA	Cohort	NA	1565	22.90 ± 2.9	2298	22.80 ± 3.2
Nishigaki et al. 2020^76^			NA		NA	530	23.20 ± 2.9		
Nishigaki et al. 2020^76^			NA		NA	723	22.90 ± 2.7		
Nishigaki et al. 2020^76^		Female	NA		NA	1592	20.40 ± 2.5	3871	20.60 ± 2.9
Nishigaki et al. 2020^76^			NA		NA	281	20.60 ± 2.4		
Nishigaki et al. 2020^76^			NA		NA	333	20.40 ± 2.4		
Agarwal et al. 2021^84^	US	Mixed	29.5	Case–control	NA	24	33.35 ± 2.9	85	35.45 ± 4.2
Agarwal et al. 2021^84^			28.6		NA	86	23.46 ± 2.0	223	22.81 ± 2.7
Bouna-Pyrrou et al. 2021^31^	Germany	Male	NA	Cross-sectional	NA	55	23.67 ± 3.5	38	22.08 ± 2.4
Bouna-Pyrrou et al. 2021^31^					NA	84	23.27 ± 2.9	9	21.43 ± 1.4
Bouna-Pyrrou et al. 2021^31^		Female	NA		NA	45	22.41 ± 2.9	54	21.60 ± 2.8
Bouna-Pyrrou et al. 2021^31^					NA	75	22.15 ± 3.2	24	22.27 ± 2.3
Gao et al. 2021^86^	China	Mixed	50.1	Cross-sectional	55.3	1016	23.50 ± 3.3	8430	23.30 ± 3.5
Hashimoto et al. 2021^87^	Japan	Mixed	62.0	Cross-sectional	15.0	3157	22.40 ± 2.9	6199	22.20 ± 3.2
Hashimoto et al. 2021^87^					29.5	1162	22.70 ± 2.9		
Hashimoto et al. 2021^87^					50.0	657	22.60 ± 2.9		
Innes et al. 2021	Great Britain	Mixed	57.0	Cohort	NA	96,388	27.30 ± 4.3	374,303	27.40 ± 4.9
Innes et al. 2021					NA	18,755	27.90 ± 4.6		
Crovetto et al. 2022^85^	Chile	Male	NA	Cross-sectional	NA	111	23.40 ± 3.1	207	23.40 ± 3.2
Crovetto et al. 2022^85^		Female			NA	266	23.10 ± 3.0	864	23.20 ± 3.1

	Correlative data								
	Country	Sex	Sample’s mean age (in years)	Study design	Mean amount of alcohol consumed (in grams per day)	n	Correlation coefficient		
Kauffmann et al. 1989^66^ ^g^	France	Male	NA	Cross-sectional	NA	384	0.14		
Ishizaki et al. 1994^65^ ^g^	Japan	Male	47.1	Cross-sectional	176.0	83	-0.01		
Kleiner et al. 2004^62^ ^g^	US	Female	40.6	Cross-sectional	NA	298	-0.12		
Gearhardt et al. 2009^g^	US	Mixed	46.6	Cohort	9.8	37,259	-0.07		
Gearhardt et al. 2009^g^						23,928	-0.16		
Gazdzinski et al. 2010^59^ ^b,h^	US	Male	50.8	Cross-sectional	NA	55	-0.07		
Gazdzinski et al. 2010^59^ ^b,h^					NA		0.17		
Gazdzinski et al. 2010^59^ ^b,h^					NA		-0.05		
Amorim et al. 2012^69^ ^g^	Brazil	Mixed	NA	Cross-sectional	NA	203	0.40		
Hooper et al. 2012^67^ ^g^	US	Mixed	13.8	Cross-sectional	NA	51	-0.04		
Hooper et al. 2012^67^ ^g^			41.8		NA	51	-0.11		
Sebelien et al. 2013^71^ ^g^	Norway	Mixed	61.0	Cross-sectional	NA	26	-0.22		
Remus et al. 2014^70^ ^g^	Romania	Mixed	20.8	Cross-sectional	NA	319	0.14		
Ventus and Jern 2016^72^ ^g^	Finland	Male	33.1	Case–control	NA	863	0.02		
Ventus and Jern 2016^72^ ^g^			43.8		NA	69	0.08		
Cronce et al. 2017^56^ ^g^	US	Female	21.3	Cross-sectional	16.2	313	0.01		
Cronce et al. 2017^56^ ^g^			20.8		13.0	425	-0.07		
Weinland et al. 2019^13^ ^h^	Germany	Male	48.0	Case–control	155.0	101	0.11		
Weinland et al. 2019^13^ ^h^		Female			60.0	72	0.10		
Kokubun et al. 2021^89^ ^g^	Japan	Mixed	49.7	Cross-sectional	NA	142	0.12		

	Odds ratio data								
	Country	Sex	Sample’s mean age (in years)	Study design	Mean amount of alcohol consumed (in grams per day)	Drinking subjects with high BMI	Drinking subjects with normal BMI	Control subjects with high BMI	Control subjects with normal BMI
						n or %	n or %	n or %	n or %
Janssens et al. 2001^75^ ^i^	Belgium	Male	45.1	Cohort	9.4	11%	89%	10%	90%
Janssens et al. 2001^75^ ^i^					27.4	13%	87%		
Janssens et al. 2001^75^ ^i^					54.0	12%	88%		
Janssens et al. 2001^75^ ^i^		Female	45.9		6.8	9%	91%	13%	87%
Janssens et al. 2001^75^ ^i^					18.8	4%	96%		
Janssens et al. 2001^75^ ^i^					36.0	9%	91%		
Wakabayashi 2012^58^ ^i^	Japan	Male	48.1	Cross-sectional	11.0	10.7%	12.4%	35.6%	27.1%
Wakabayashi 2012^58^ ^i^					33.0	32.8%	37.4%		
Wakabayashi 2012^58^ ^i^					66.0	20.9%	23.0%		
Santangelo et al. 2019^73^	Italy	Mixed	22.1	Cross-sectional	NA	27	104	183	673
Weinland et al. 2019^13^ ^j^	Germany	Male	48.0	Case–control	155.0	NA	NA	NA	NA
Weinland et al. 2019^13^ ^j^		Female			60.0	NA	NA	NA	NA

^a^Data obtained from graphs.

^b^Data obtained by contacting the authors.

^c^Excluded as outlier in sensitivity analyses.

^d^Standard deviations derived from confidence intervals.

^e^Standard deviations derived from standard errors of the mean.

^f^Standard deviations derived from p-values.

^g^Pearson correlation coefficient r.

^h^Spearman’s ρ.

^i^No absolute numbers reported.

^j^Data only reported as odds ratios.

### Meta-analytic results and moderator analyses

**Figure 2:**
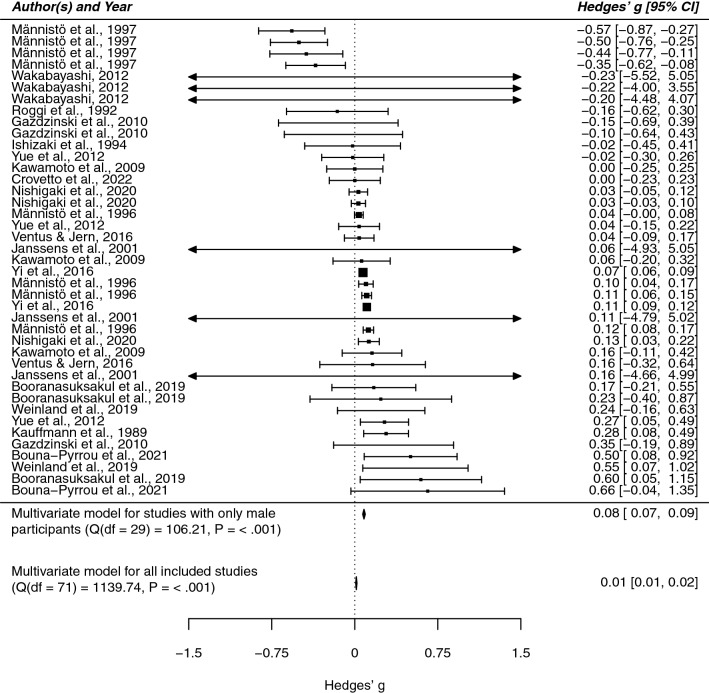
Forest plot of the standardized mean difference in BMI among male drinking and non-drinking subjects. This plot shows the results of the individual studies examining males together with their 95% confidence interval (CI). The weight of each study contributing to the overall effect is illustrated by the size of the square. The summary polygons at the bottom of the plot show the results from the multivariate meta-analytic model for (1) the subgroup of males and for (2) all included studies.

**Figure 3:**
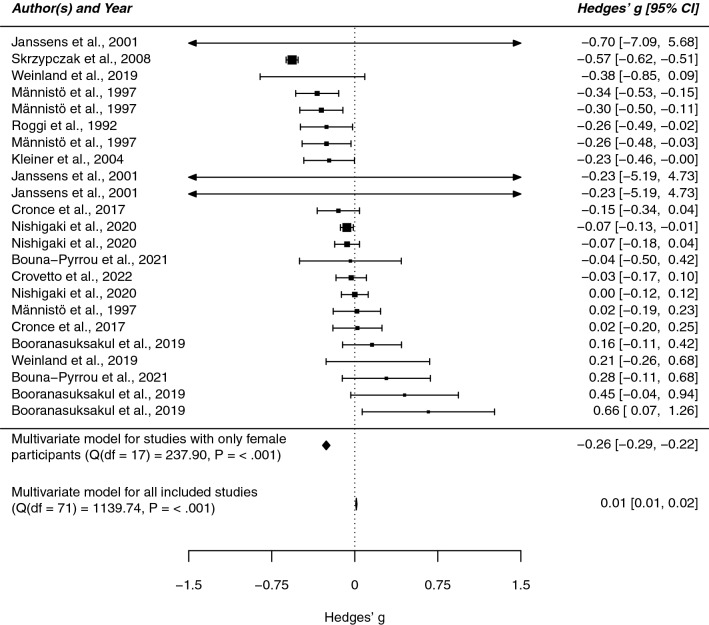
Forest plot of the standardized mean difference in BMI among female drinking and non-drinking subjects. This plot shows the results of the individual studies examining females together with their 95% confidence interval (CI). The weight of each study contributing to the overall effect is illustrated by the size of the square. The summary polygons at the bottom of the plot show the results from the multivariate meta-analytic model for (1) the subgroup of females and for (2) all included studies.

Since sensitivity analyses revealed one substantially influential outlier^78^, we conducted our analysis without this study. When analyzing all of the studies together we found a very small positive association between alcohol use and BMI (g = 0.01, 95% CI [0.01; 0.02]) with a high heterogeneity index (Q = 1139.74, p < 0.0001). Including the factor sex as a moderator revealed a significant moderating effect for male (z = 0.08, p < 0.0001) and female sex (z = − 0.26, p < 0.0001) while the group of studies with unknown sex distribution remained non-significant (z = 0.004, p = 0.27). This indicates that sex substantially influences the effect size and we therefore conducted sex-separated analyses: while in men the association was slightly positive (g = 0.08, 95% CI [0.07; 0.09]), we found a negative effect in women (g = − 0.26, 95% CI [− 0.29; − 0.22]). In both samples, heterogeneity was rather high (Q = 237.90 for women, Q = 106.21 for men, p < 0.0001 for both) indicating that these models are not yet of adequate fit for the data and require further moderator analyses. The sex-separated results in comparison to the overall results are shown in Figs. 2 and 3.

Bearing this sex-diverging result in mind, we first analyzed the interaction of sex and ethnicity before conducting a subgroup analysis for this moderator. It revealed a significant interaction of Asian ethnicity with female sex (z = 0.39, p < 0.0001) again suggesting that analyses concerning ethnicity should be performed sex-separately. No African and only one Hispanic study provided sex-separated data; therefore, sex-separated subgroup analyses were limited to Asian vs. Caucasian participants. In men, these ethnic groups did not differ significantly (z = 1.35, p = 0.51), whereas in women, the negative association between alcohol consumption and BMI was more pronounced in Caucasian than in Asian participants (g = − 0.45 compared to g = − 0.05; z = 11.5, p < 0.0001).

Due to insufficient data, it was not possible to compute the moderator analysis distinguishing between different beverage types: only one study^64^ reported separate BMI data for beer, wine, and liquor drinkers.

### Meta-regression analyses

Since all our meta-analytic results revealed a strong influence of sex, we also performed sex-separated meta-regression analyses. The threshold for significant results was Bonferroni-corrected at p = 0.006.

The meta-regression analysis concerning the amount of alcohol in gram per day revealed a significant moderating influence on the effect sizes of the overall sample (β = 0.0009, p < 0.0001) and of men (β = 0.001, p < 0.0001). This result suggests that in men with every additional gram of alcohol per day the association of alcohol use and BMI increases by 0.001. In women, the meta-regression was not significant (β = 0.002, p = 0.07). The addition of a quadratic term did not explain for more variance than the linear term (no U-shaped association detectable) (data not shown). In our sample, the mean alcohol consumption was 23.14 g/day for men and 13.82 g/day for women.

Ethanol intake and smoking was correlated in our sample, insofar that the amount of alcohol (g/day) and the percentage of smokers in the drinking group was moderately associated for males (r = 0.39, p = 0.097) and highly associated for females (r = 0.92, p = 0.003). The meta-regression regarding the percentage of smokers revealed a non-significant influence on the effect size (males: β = 0.06, p = 0.05; females: β = − 0.52, p = 0.09).

The mean study quality assessed via the Newcastle–Ottawa-Scale^39,40^ was 5.37 ± 0.79. The meta-regression analyses regarding study quality remained non-significant for the male sample (β = − 0.019, p = 0.11), but suggest an influence of the studies’ risk of bias on the female (β = 0.350, p < 0.0001) and the overall effect size (β = − 0.038, p < 0.0001).

### Small study effects and sensitivity analyses

When visually examining the funnel plot (Supplementary Fig. S1), no evidence of small study effects or publication bias was detectable. Sensitivity analyses revealed one influential outlier^78^ whose inclusion biased the results substantially, especially in terms of heterogeneity (result before exclusion: g = − 0.04, 95% CI [− 0.05, − 0.04], Q = 115,516.38; after exclusion: g = 0.01, 95% CI [0.01; 0.02], Q = 1139.74). Therefore, this study was excluded from all analyses.

## Discussion

To our knowledge, this is the first systematic literature search and meta-analysis to investigate how alcohol use associates with BMI in normal to overweight individuals while focusing on possible sex differences. We found a sex-diverging relationship of small size: whereas alcohol use was slightly related to higher BMI in men, it was more strongly linked to lower BMI in women. Thus, our meta-analytic findings provide empirical evidence to confirm the previous assumption of a systematic review^29^ that alcohol use is to a small extent, but positively associated with BMI in men; to this state of knowledge, we also add that alcohol use in women is related to a lower BMI. This also matches data of a recent study indicating that higher BMI is related to an increased risk for hospital readmissions in male in-patients with AUD, while it tends to be protective in female in-patients^90^. When analyzing both sexes together, our results match previous meta-analyses suggesting that higher alcohol intake associates with higher BMI^34,35^. In terms of practical relevance, the small effect sizes of up to Hedges’ g = − 0.26 mean that the BMI of approximately 60% of female drinkers is below the average BMI of non-drinking women^91^. One has to bear in mind that with a non-overlap of approximately 15% between drinkers and non-drinkers the practical impact of these findings is small^91^. Nevertheless, these effects raise the question which mechanisms underlie the sex-diverging relationships between alcohol use and BMI. In moderator analyses, we found significant and sex-separated effects of the amount of daily ethanol intake and ethnicity.

The amount of alcohol consumed daily influenced the BMI in men. This supports the assumption that caloric intake due to alcohol consumption leads to higher BMI in men. Sayon-Orea et al.^29^ suggest in their review that the caloric impact of alcohol use (represented by a positive association of BMI and alcohol use) is only evident in subjects who drink more often and in larger quantity. On average, males consume more alcohol than females^1^ which also holds true for the here analyzed sample (mean[males] = 23.14 g/day; mean[females] = 13.82 g/day). It is possible that a part of the sex-diverging effect found here is attributable to the differences in the mean ethanol intake between men and women. Accordingly, we did not observe such a significant moderating effect in the female samples, which suggests that either their drinking quantity was too low or that mechanisms other than calorie supply are relevant in women. A further explanation of the positive association found in men might be the toxic effect of alcohol on different body functions. Regular alcohol consumption can lead to a state of generalized insulin resistance by inhibiting, for example, glucose disposal or insulin release in men^17^. This resistance is commonly paralleled by higher body weight^18,92^.

We aimed at testing whether sex differences in choice of beverage type account for the sex diverging relationships of alcohol use and BMI. However, we were not able to provide meta-analytic support of this association here, as our systematic literature search identified only one early paper that provided beverage type-specific data^64^. This study indicates higher BMI in liquor than in wine drinkers of both sexes. Similarly, Sayon-Orea et al.^29^ found that beer and liquor consumption (≥ 7 drinks/week) are associated with weight gain, whereas no such effect was found for wine consumption. We also analyzed correlations between beverage-type specific alcohol drinking and BMI in an additional data set^7^ and found a positive correlation between liquor intake and BMI in male patients with AUD (see Supplementary Fig. S2). As men prefer liquor^7,93^, the higher caloric intake associated with liquor consumption (which is more often found in men vs. women) might account for our observation of higher BMI in men with alcohol use vs. men who deny alcohol consumption. Furthermore, the sex-specific choice of beverage type might also help to explain why our meta-analysis demonstrates lower BMI in women with alcohol use. Consumption of wine, but not use of beer or liquor, is related to more frequent exercising in both sexes^94^ and to a more healthy dietary behavior^95,96^. Choice of beverage type was also suggested to associate with intake of fat, carbohydrate, and vitamins^64^. Women more often choose wine^7,93^ and thus the alcohol intake in women is expected to be related to more frequent exercising and possibly to a more healthy life-style which might account for the here observed lower BMI in alcohol-using women. However, future research is needed to study the mechanisms underlying the identified sex-diverging association between alcohol use and BMI.

A higher percentage of smokers in the drinking group amplified the sex-diverging association of alcohol use and BMI but remained non-significant. Usually, smoking is associated with weight loss and lower BMI^80,97^ as in our female sample. However, more recent studies with large sample sizes suggest that this relationship is less clear, especially in obese persons^98,99^, corresponding to the results in our male sample. Weinland et al.^13^ also reported an amplifying effect of active smoking status which might be due to a positive association of smoking and alcohol consumption in general^61,80^. In our study, these two variables were highly correlated, as well. It is possible that the results of this meta-regression reflect the results for subjects drinking in higher quantity since subgroups comprising more smokers consumed more alcohol here.

We here also provide first meta-analytic evidence that ethnicity modulates the sex-separated relationship between alcohol use and BMI. The effect size of the lower BMI values in alcohol-drinking vs. control women was stronger in the Caucasian than in the Asian subsample. It is well-established that genetics influences the response to alcohol and the vulnerability to develop AUD^100,101^, and this might explain the here observed inter-ethnic variation of the relationship between alcohol use and BMI.

### Limitations and strengths

There are some limitations to this study. First, the literature search was restricted to titles resulting in a smaller number of eligible studies. We tried to extend the literature search to abstracts or a full-text search, but the number of results was too high to be economically screened for eligibility. Additionally, the literature search only identified two studies addressing patients with AUD^13,59^; thus, it remains to be shown whether the here reported associations could be generalized to AUD. Third, we could not properly test for an inverted U-shaped relationship between the amount of alcohol consumption and BMI since we lacked studies with highly consuming participants. Even if there was an inverted U-shaped association between these two variables, our dataset with light to moderate drinkers would only be able to detect the ascending slope up to the point of inflection. Fourth, all effect sizes computed in this study are small following Cohen^102^. Consequently, they do not reach the extent of the minimal clinically relevant difference defined by Sayon-Orea et al.^29^. Fifth, the usage of a multivariate meta-analytic model might also involve some limitations^103^. It assumes, for example, that missing values are missing at random which is not always true, especially when they are missing due to non-significance. Furthermore, additional modeling assumptions are required and harder to verify than in univariate meta-analyses. Applying the multivariate model also entails numerous strengths. It uses more information improving statistical properties such as smaller mean-square error and greater precision^104^. It also allows for accounting for covariances between pooled estimates and for reducing reporting bias when some outcomes are selectively missing. A detailed summary of advantages and disadvantages of multivariate meta-analysis can be found in Jackson et al.^103^. Further strengths of this study are the strict adherence to the standardized PRISMA guidelines^36,37^ (see Supplementary Table S2), the probable absence of small study effects, the reasonably robust results revealed by our sensitivity analyses and by the stability of our results after updating the literature search, and the rather representative control group. In case–control studies, a control group is usually defined by the strict absence of the variable of interest^105^. Concerning the variable alcohol use, this is not typical for the average population where, for example, only 23.5% are lifetime abstainers in the European region^1^. Our control subjects are defined by different measures (see section “Statistical analyses”) and therefore, are not classed among ”super healthy” controls. Thus, our results can more easily be generalized to the general population^105^. This also holds true for our drinking group with especially women being comparable to the general population in terms of their mean ethanol intake (14 g/day here compared to 15 g/day among females worldwide^1^). Additionally, this is, up to our knowledge, the first meta-analysis specifically examining the association of alcohol consumption and BMI for different ethnicities.

## Conclusion

As far as we know, this is the first meta-analysis to show that alcohol use associates with higher BMI in men and lower BMI in women. The effect size is lower in female Asians than Caucasians and it is influenced by the amount of alcohol consumption per day. However, our effect sizes did not reach the extent of the minimal clinically relevant difference defined by a previous review^29^. The differences in average daily alcohol consumption between men and women as well as sex-specific choice of beverage linked to life style factors such as exercise and intake of fat and carbohydrates might account for these sex-diverging effects. This interpretation requests validation by future studies. Our findings lay the foundation of further studies investigating mechanisms underlying alcohol use.

## Supplementary Information

Supplementary Information.

## Data Availability

The datasets generated during and/or analysed during the current study are available from the corresponding author on reasonable request.
