# Supplementary material for: Meta-analytic evidence for a sex-diverging association between alcohol use and body mass index
Source: Sci Rep. 2022 Dec 19;12:21869. doi: 10.1038/s41598-022-25653-w (PMC9763242; doi:10.1038/s41598-022-25653-w)
Supplement: Supplementary file 1 — Supplementary Information. [file 41598_2022_25653_MOESM1_ESM.docx]

**ONLINE SUPPLEMENT**

**FOR**

**Meta-Analytic Evidence for a Sex-Diverging Association Between Alcohol Use and Body Mass Index**

*A Systematic Review and Meta-Analysis of Case-control, Cohort, and Cross-sectional studies*

Eva-Maria Siegmann^1,^*, Massimiliano Mazza^2^, Christian Weinland^1^, Falk Kiefer^2^, Johannes Kornhuber^1^, Christiane Mühle^1,#^, Bernd Lenz^2,#^

^1^Department of Psychiatry and Psychotherapy, Friedrich-Alexander University Erlangen-Nürnberg (FAU), Germany.

^2^Department of Addictive Behavior and Addiction Medicine, Central Institute of Mental Health (CIMH), Medical Faculty Mannheim, Heidelberg University, Germany.

*Department of Psychiatry and Psychotherapy, Friedrich-Alexander University Erlangen-Nürnberg (FAU), Schwabachanlage 6, 91054 Erlangen, Germany

Phone: +49 9131 85-34166, Fax: +49 9131 85-36002

E-Mail: eva-maria.siegmann@uk-erlangen.de

^#^These authors contributed equally.

**Table of content**

[Supplementary Figure S1. Funnel plot with all included studies 3](#_Toc116895346)

[Supplementary Figure S2. Association of BMI and liquor consumption since onset of regular drinking 4](#_Toc116895347)

[Supplementary Table S1. The coding protocol 6](#_Toc116895348)

[Supplementary Table S2. PRISMA checklist 13](#_Toc116895349)

# Supplementary Figure S1. Funnel plot with all included studies


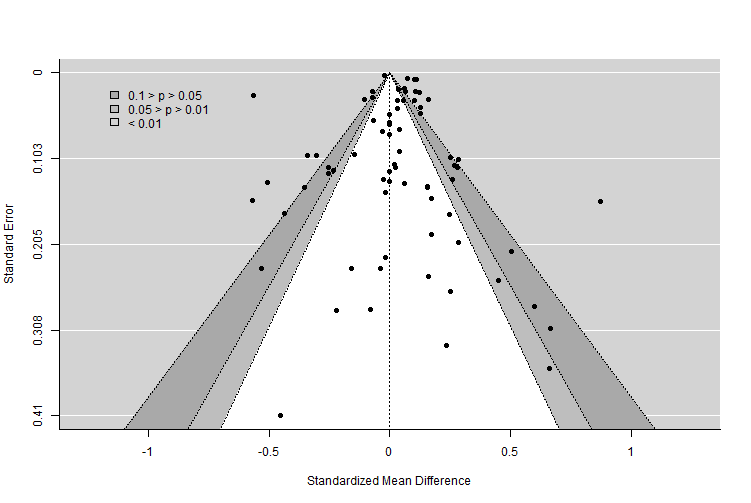


# Supplementary Figure S2. Association of BMI and liquor consumption since onset of regular drinking


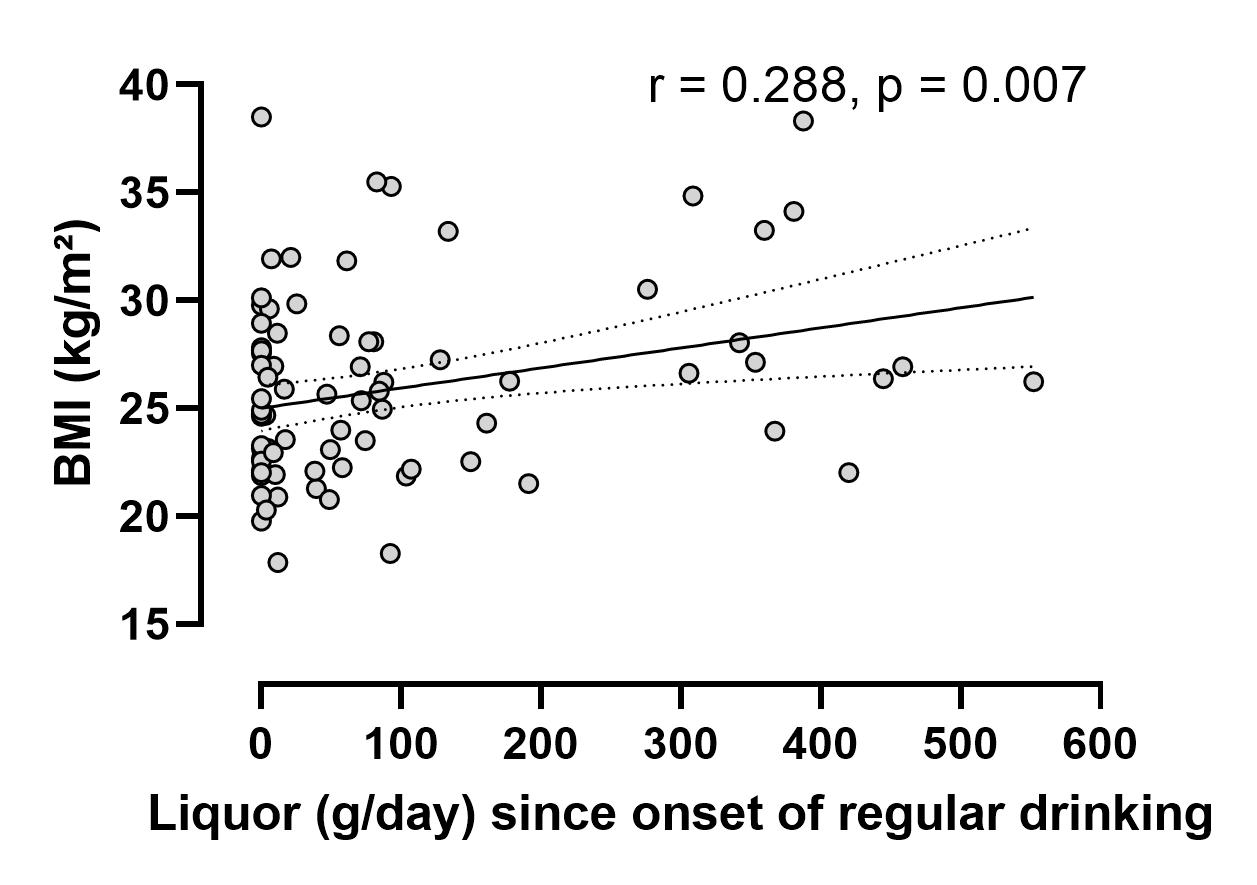


More daily liquor consumption since onset of regular alcohol drinking correlates with higher BMI in male patients with alcohol use disorder (Pearson correlation, n = 88, r = 0.288, 95% confidence interval from bias-corrected and accelerated bootstrap (1000 resamples) analysis 0.071 – 0.477, p = 0.007). There were no other significant correlations of BMI with daily beer or wine consumption in male or female patients or with daily liquor consumption since onset of regular dinking in female patients (data not shown). We here analyzed data from the Neurobiology of Alcoholism Study. In short, we recruited 113 male and 87 female patients with alcohol use disorder and measured body mass index (BMI) and lifetime alcohol drinking (Lifetime Drinking History interview [1]) (for more details see previous publications [2-16]).

References:

1. Skinner, H. A. & Sheu, W. J. Reliability of alcohol use indices. The Lifetime Drinking History and the MAST. *Journal of studies on alcohol* **43,** 1157–1170; 10.15288/jsa.1982.43.1157 (1982).

2. Weinland, C., Tanovska, P., Kornhuber, J., Mühle, C. & Lenz, B. Serum lipids, leptin, and soluble leptin receptor in alcohol dependence: A cross-sectional and longitudinal study. *Drug and alcohol dependence* **209,** 107898; 10.1016/j.drugalcdep.2020.107898 (2020).

3. Weinland, C., Mühle, C., Zimmermann, C. von, Kornhuber, J. & Lenz, B. Sulphated dehydroepiandrosterone serum levels are reduced in women with alcohol use disorder and correlate negatively with craving: A sex-separated cross-sectional and longitudinal study. *Addiction Biology* **27,** e13135; 10.1111/adb.13135 (2022).

4. Weinland, C., Mühle, C., Kornhuber, J. & Lenz, B. Progesterone serum levels correlate negatively with craving in female postmenopausal in-patients with alcohol use disorder: A sex- and menopausal status-separated study. *Progress in neuro-psychopharmacology & biological psychiatry* **110,** 110278; 10.1016/j.pnpbp.2021.110278 (2021).

5. Weinland, C., Mühle, C., Kornhuber, J. & Lenz, B. Crossed Eye/Hand Laterality and Left-Eyedness Predict a Positive 24-Month Outcome in Alcohol-Dependent Patients. *Alcoholism: Clinical and Experimental Research* **43,** 1308–1317; 10.1111/acer.14051 (2019).

6. Weinland, C., Mühle, C., Kornhuber, J. & Lenz, B. Body mass index and craving predict 24-month hospital readmissions of alcohol-dependent in-patients following withdrawal. *Progress in neuro-psychopharmacology & biological psychiatry* **90,** 300–307; 10.1016/j.pnpbp.2018.11.019 (2019).

7. Weinland, C., Braun, B., Mühle, C., Kornhuber, J. & Lenz, B. Cloninger Type 2 Score and Lesch Typology Predict Hospital Readmission of Female and Male Alcohol-Dependent Inpatients During a 24-Month Follow-Up. *Alcoholism: Clinical and Experimental Research* **41,** 1760–1767; 10.1111/acer.13468 (2017).

8. Müller, C. P., Mühle, C., Kornhuber, J. & Lenz, B. Sex-Dependent Alcohol Instrumentalization Goals in Non-Addicted Alcohol Consumers versus Patients with Alcohol Use Disorder: Longitudinal Change and Outcome Prediction. *Alcoholism: Clinical and Experimental Research* **45,** 577–586; 10.1111/acer.14550 (2021).

9. Mühle, C., Barry, B., Weinland, C., Kornhuber, J. & Lenz, B. Estrogen receptor 1 gene variants and estradiol activities in alcohol dependence. *Progress in neuro-psychopharmacology & biological psychiatry* **92,** 301–307; 10.1016/j.pnpbp.2019.01.008 (2019).

10. Mühle, C., Weinland, C., Gulbins, E., Lenz, B. & Kornhuber, J. Peripheral Acid Sphingomyelinase Activity Is Associated with Biomarkers and Phenotypes of Alcohol Use and Dependence in Patients and Healthy Controls. *International Journal of Molecular Sciences* **19,** 4028; 10.3390/ijms19124028 (2018).

11. Lenz, B. *et al.* Oxytocin blood concentrations in alcohol use disorder: A cross-sectional, longitudinal, and sex-separated study. *European neuropsychopharmacology : the journal of the European College of Neuropsychopharmacology* **51,** 55–67; 10.1016/j.euroneuro.2021.04.015 (2021).

12. Lenz, B. *et al.* Prenatal and adult androgen activities in alcohol dependence. *Acta Psychiatrica Scandinavica* **136,** 96–107; 10.1111/acps.12725 (2017).

13. Lenz, B., Köllner, M. G., Mühle, C., Weinland, C. & Kornhuber, J. Basic Human Body Dimensions Relate to Alcohol Dependence and Predict Hospital Readmission. *Journal of Clinical Medicine* **8,** 2076; 10.3390/jcm8122076 (2019).

14. Kalinichenko, L. S. *et al.* Neutral sphingomyelinase mediates the co-morbidity trias of alcohol abuse, major depression and bone defects. *Molecular psychiatry* **26,** 7403–7416; 10.1038/s41380-021-01304-w (2021).

15. Gegenhuber, B., Weinland, C., Kornhuber, J., Mühle, C. & Lenz, B. OPRM1 A118G and serum β-endorphin interact with sex and digit ratio (2D:4D) to influence risk and course of alcohol dependence. *European neuropsychopharmacology : the journal of the European College of Neuropsychopharmacology* **28,** 1418–1428; 10.1016/j.euroneuro.2018.09.002 (2018).

16. Braun, B., Weinland, C., Kornhuber, J. & Lenz, B. Religiosity, Guilt, Altruism and Forgiveness in Alcohol Dependence: Results of a Cross-sectional and Prospective Cohort Study. *Alcohol and alcoholism (Oxford, Oxfordshire)* **53,** 426–434; 10.1093/alcalc/agy026 (2018).

# Supplementary Table S1. The coding protocol

**Coding protocol**

*BMI and alcohol consumption*

**Inclusion criteria:**

1. Publication language: English
2. Body mass index was measured as continuous measure.
3. Cases (alcohol consuming or alcohol addicted persons) and controls were compared concerning their body mass index OR the correlation of body mass index and any form of alcohol consumption (measured continuously) was examined OR both analyses were applied.
4. In case-control studies, controls were either not diagnosed with any form of alcohol use disorder or were not drinking alcohol at all.
5. Effect sizes or associated data to compute effect sizes are reported.

**Exclusion criteria:**

1. Abstracts or preliminary data
2. Publication language other than English
3. Body mass index was measured as a categorical measure
4. Only partial correlations or β-coefficients from multiple regression models are reported

**Coding procedure:**

1. One line represents one effect size
2. If effect sizes are reported separately for the whole sample and for subgroups, predominantly information concerning the subgroups will be extracted. Every subgroup will be treated as distinct sample (variable sno), unless they are compared to the same control group.
3. If there are multiple effect sizes within one sample concerning different outcome variables (e.g. different ways of operationalizing alcohol consumption), every effect size will be reported in its own line. These effect sizes are coded as dependent by allocating the same number for the variable sno.
4. No computations should be carried out while coding. Information is extracted directly without conversions.

| **Variable** | **Description** | | | | **Code** | **Example** |  |
| --- | --- | --- | --- | --- | --- | --- | --- |
| ***General and sample characteristics*** | | | | | | |  |
| study | Study name comprising lead author and year of publication. | | | | Free specification | Meyer2000  Meyer2000a |  |
| pubyear | Year of publication | | | | Range: [1950, 2021] | 2007 |  |
| incl | Effect size can or cannot be included in statistical analysis | | | | 0 = exclude  1 = include | 1 |  |
| sid | Consecutive number for every publication | | | | Range: [1, ∞] | 1 |  |
| sno | Consecutive number for every sample | | | | Range: [1, ∞] | 1 |  |
| colyear | Year of conduct | | | | Range: [1950, 2021] | 2007 |  |
| cntry | Country of conduct  If not reported, extract affiliation of lead author as ISO-CODE 2:  <http://en.wikipedia.org/wiki/ISO_3166-1_alpha-2>  or „XX“ describing samples with participants originating from different  countries | | | | Free specification | DE |  |
| pubtype | Publication type | | | | 1 = Peer-reviewed Journal  2 = Book  3 = Thesis (Master / PhD)  4 = Poster  5 = Other | 1 |  |
| n | Sample size *N* | | | | Range: [2, ∞] | 100 |  |
| samtype | Description of sample (coded) | | | | 1 = Youth  2 = Adults, mixed  3 = Students  (undergraduates, college)  sample | 2 |  |
| age | Mean age (in years) | | | | Range: [0, ∞] | 16.86 |  |
|  |  | | | |  |  |  |
| **Variable** | | **Description** | **Code** | | | **Example** | |
| ***Effect sizes***  ***(1 = affected group; 2 = control group)*** | | | | | | | |
| n1 | | Sample size of drinking group | | Range: [2, ∞] | | 100 | |
| m1 | | Mean BMI of drinking group | | Range: [0, ∞] | | 0.9 | |
| sd1 | | Standard deviation of BMI of drinking group | | Range: [0, ∞] | | 0.1 | |
| sem1 | | Standard error of the mean of BMI of drinking group | | Range: [0, ∞] | | 0.1 | |
| cilow1 | | Lower limit of the 95% confidence interval reported for mean BMI of the drinking group | | Range: [0, ∞] | | 0.8 | |
| ciup1 | | Upper limit of the 95% confidence interval reported for mean BMI of the drinking group | | Range: [0, ∞] | | 1.0 | |
| n2 | | Sample size of non-drinking group | | Range: [2, ∞] | | 100 | |
| m2 | | Mean BMI of non-drinking group | | Range: [0, ∞] | | 0.9 | |
| sd2 | | Standard deviation of BMI of non-drinking group | | Range: [0, ∞] | | 0.1 | |
| sem2 | | Standard error of the mean of BMI of non-drinking group | | Range: [0, ∞] | | 0.1 | |
| cilow2 | | Lower limit of the 95% confidence interval reported for mean BMI of the non-drinking group | | Range: [0, ∞] | | 0.8 | |
| ciup2 | | Upper limit of the 95% confidence interval reported for mean BMI of the non-drinking group | | Range: [0, ∞] | | 1.0 | |
| pval | | P-value corresponding to a (t-)test of mean BMI difference | | Range: [0, 1] | | 0.5 | |
| tval | | T-test-value comparing two BMI means | | Range: [-∞ , ∞] | | 1.0 | |
| sign | | **Only in the case of p-value-data**: Indicator variable whether the first or the second group had the lower mean | | 1 = m1 > m2  -1 = m1 < m2 | | 1 | |
| n_r | | **Only in the case of correlative data**: Sample size related to the correlative analysis | | Range: [2, ∞] | | 100 | |
| r | | **Only in the case of correlative data**: Pearson’s correlation coefficient r | | Range: [-1, 1] | | 0.5 | |
| **Variable** | | **Description** | | **Code** | | **Example** | |
| ***Effect sizes***  ***(1 = affected group; 2 = control group)*** | | | | | | | |
| rho | | **Only in the case of correlative data**: Spearman’s correlation coefficient rho | | Range: [-1, 1] | | 0.5 | |
| cell1 | | **Only in the case of odds-ratio-data**: Proportion or number of drinking subjects with increased BMI | | Range: [0, ∞] | | 10 | |
| cell2 | | **Only in the case of odds-ratio-data**: Proportion or number of drinking subjects with normal BMI | | Range: [0, ∞] | | 10 | |
| cell3 | | **Only in the case of odds-ratio-data**: Proportion or number of non-drinking subjects with increased BMI | | Range: [0, ∞] | | 10 | |
| cell4 | | **Only in the case of odds-ratio-data**: Proportion or number of non-drinking subjects with normal BMI | | Range: [0, ∞] | | 10 | |
| or | | **Only in the case of odds-ratio-data**: Odds ratio | | Range: [-∞ , ∞] | | 1.0 | |

| **Variable** | **Description** | **Code** | | **Example** |
| --- | --- | --- | --- | --- |
| ***Additional information*** | | | | |
| qual1 | Category Selection   - Is the case definition adequate? - Representativeness of cases - Selection of controls - Definition of controls | | 0 = 0 Stars  1 = 1 Star  2 = 2 Stars  3 = 3 Stars  4 = 4 Stars | 1 |
| qual2 | Category Comparability: Comparability of cases and controls on the basis of the design or analysis   - Study controls for gender - Study controls for any additional factor | | 0 = 0 Stars  1 = 1 Star  2 = 2 Stars | 1 |
| qual3 | Category Exposure   - Ascertainment of exposure - Same method of ascertainment for cases and controls - Non-response rate | | 0 = 0 Stars  1 = 1 Star  2 = 2 Stars  3 = 3 Stars | 1 |
| qual_r1 | **Only in the case of correlative data:** Category Selection   - Representativeness of the sample - Sample size - Ascertainment of exposure - Non-respondents | | 0 = 0 Stars  1 = 1 Star  2 = 2 Stars  3 = 3 Stars  4 = 4 Stars | 1 |
| qual_r2 | **Only in the case of correlative data:** Category Comparability: Confounding factors are controlled   - The study controls for the most important factor = gender - The study controls for any additional factor | | 0 = 0 Stars  1 = 1 Star  2 = 2 Stars | 1 |
| qual_r3 | **Only in the case of correlative data:** Category Outcome:   - Assessment of Outcome - The statistical test used to analyze the data is clearly described and appropriate | | 0 = 0 Stars  1 = 1 Star  2 = 2 Stars | 1 |

| **Variable** | **Description** | **Code** | | **Example** |
| --- | --- | --- | --- | --- |
| ***Additional information*** | | | | |
| gender | Gender of examined sample | | 1 = male  2 = female  3 = mixed sample | 1 |
| addict | Does the examined affected group consist of alcohol-addicted subjects? | | 0 = no  1 = yes | 1 |
| smoke | Percentage of current smokers in the drinking group | | Range: [0, 1] | 0.5 |
| ethnic | Predominant ethnicity of examined sample | | 1 = White-Caucasian  2 = Asian  3 = African  4 = Hispanic / South America | 1 |
| design | Study design: Case-control vs. cross-sectional / cohort study | | 1 = case-control  2 = cohort or cross- sectional | 1 |
| drink | Type of beverage the drinking group consumes | | 1 = Beer  2 = Wine  3 = Spirits  4 = Mixed | 1 |
| amount | Average alcohol consumption in grams per day | | Range: [0, ∞] | 10 |

# Supplementary Table S2. PRISMA checklist

| **Section and Topic** | **Item #** | **Checklist item** | **Location where item is reported** |
| --- | --- | --- | --- |
| **TITLE** | | |  |
| Title | 1 | Identify the report as a systematic review. | Title page |
| **ABSTRACT** | | |  |
| Abstract | 2 | See the PRISMA 2020 for Abstracts checklist. | Abstract |
| **INTRODUCTION** | | |  |
| Rationale | 3 | Describe the rationale for the review in the context of existing knowledge. | ll. 62-75 |
| Objectives | 4 | Provide an explicit statement of the objective(s) or question(s) the review addresses. | ll. 72-75 |
| **METHODS** | | |  |
| Eligibility criteria | 5 | Specify the inclusion and exclusion criteria for the review and how studies were grouped for the syntheses. | Table S1 |
| Information sources | 6 | Specify all databases, registers, websites, organisations, reference lists and other sources searched or consulted to identify studies. Specify the date when each source was last searched or consulted. | ll. 79-90 |
| Search strategy | 7 | Present the full search strategies for all databases, registers and websites, including any filters and limits used. | ll. 79-90 |
| Selection process | 8 | Specify the methods used to decide whether a study met the inclusion criteria of the review, including how many reviewers screened each record and each report retrieved, whether they worked independently, and if applicable, details of automation tools used in the process. | ll. 93-100 |
| Data collection process | 9 | Specify the methods used to collect data from reports, including how many reviewers collected data from each report, whether they worked independently, any processes for obtaining or confirming data from study investigators, and if applicable, details of automation tools used in the process. | ll. 93-100 |
| Data items | 10a | List and define all outcomes for which data were sought. Specify whether all results that were compatible with each outcome domain in each study were sought (e.g. for all measures, time points, analyses), and if not, the methods used to decide which results to collect. | Table S1 |
|  | 10b | List and define all other variables for which data were sought (e.g. participant and intervention characteristics, funding sources). Describe any assumptions made about any missing or unclear information. | Table S1 |
| Study risk of bias assessment | 11 | Specify the methods used to assess risk of bias in the included studies, including details of the tool(s) used, how many reviewers assessed each study and whether they worked independently, and if applicable, details of automation tools used in the process. | ll. 93-100 |
| Effect measures | 12 | Specify for each outcome the effect measure(s) (e.g. risk ratio, mean difference) used in the synthesis or presentation of results. | ll. 106-109 |
| Synthesis methods | 13a | Describe the processes used to decide which studies were eligible for each synthesis (e.g. tabulating the study intervention characteristics and comparing against the planned groups for each synthesis (item #5)). | ll. 93-100 |
|  | 13b | Describe any methods required to prepare the data for presentation or synthesis, such as handling of missing summary statistics, or data conversions. | ll. 110-117 |
|  | 13c | Describe any methods used to tabulate or visually display results of individual studies and syntheses. | Table S2 |
|  | 13d | Describe any methods used to synthesize results and provide a rationale for the choice(s). If meta-analysis was performed, describe the model(s), method(s) to identify the presence and extent of statistical heterogeneity, and software package(s) used. | ll. 103-137 |
|  | 13e | Describe any methods used to explore possible causes of heterogeneity among study results (e.g. subgroup analysis, meta-regression). | ll. 124-133 |
|  | 13f | Describe any sensitivity analyses conducted to assess robustness of the synthesized results. | ll. 134-137 |
| Reporting bias assessment | 14 | Describe any methods used to assess risk of bias due to missing results in a synthesis (arising from reporting biases). | ll. 96-100, Table S1 |
| Certainty assessment | 15 | Describe any methods used to assess certainty (or confidence) in the body of evidence for an outcome. | ll. 96-100, Table S1 |
| **RESULTS** | | |  |
| Study selection | 16a | Describe the results of the search and selection process, from the number of records identified in the search to the number of studies included in the review, ideally using a flow diagram. | Figure S1, ll. 141-143 |
|  | 16b | Cite studies that might appear to meet the inclusion criteria, but which were excluded, and explain why they were excluded. | Figure S1 |
| Study characteristics | 17 | Cite each included study and present its characteristics. | Table S2 |
| Risk of bias in studies | 18 | Present assessments of risk of bias for each included study. |  |
| Results of individual studies | 19 | For all outcomes, present, for each study: (a) summary statistics for each group (where appropriate) and (b) an effect estimate and its precision (e.g. confidence/credible interval), ideally using structured tables or plots. | Table S2, Figure 1 + 2 |
| Results of syntheses | 20a | For each synthesis, briefly summarise the characteristics and risk of bias among contributing studies. | Table S2 |
|  | 20b | Present results of all statistical syntheses conducted. If meta-analysis was done, present for each the summary estimate and its precision (e.g. confidence/credible interval) and measures of statistical heterogeneity. If comparing groups, describe the direction of the effect. | Figure 1+2, ll. 146-156 |
|  | 20c | Present results of all investigations of possible causes of heterogeneity among study results. | ll. 146-186 |
|  | 20d | Present results of all sensitivity analyses conducted to assess the robustness of the synthesized results. | ll. 189-192 |
| Reporting biases | 21 | Present assessments of risk of bias due to missing results (arising from reporting biases) for each synthesis assessed. | ll. 184-186 |
| Certainty of evidence | 22 | Present assessments of certainty (or confidence) in the body of evidence for each outcome assessed. | ll. 184-192 |
| **DISCUSSION** | | |  |
| Discussion | 23a | Provide a general interpretation of the results in the context of other evidence. | ll. 204-244 |
|  | 23b | Discuss any limitations of the evidence included in the review. | ll. 253-267 |
|  | 23c | Discuss any limitations of the review processes used. | ll. 253-267 |
|  | 23d | Discuss implications of the results for practice, policy, and future research. | ll. 287-292 |
| **OTHER INFORMATION** | | |  |
| Registration and protocol | 24a | Provide registration information for the review, including register name and registration number, or state that the review was not registered. |  |
|  | 24b | Indicate where the review protocol can be accessed, or state that a protocol was not prepared. |  |
|  | 24c | Describe and explain any amendments to information provided at registration or in the protocol. |  |
| Support | 25 | Describe sources of financial or non-financial support for the review, and the role of the funders or sponsors in the review. | Additional information |
| Competing interests | 26 | Declare any competing interests of review authors. | Additional information |
| Availability of data, code and other materials | 27 | Report which of the following are publicly available and where they can be found: template data collection forms; data extracted from included studies; data used for all analyses; analytic code; any other materials used in the review. | Data availability statement |
